# Functional Analysis of a Wheat AGPase Plastidial Small Subunit with a Truncated Transit Peptide

**DOI:** 10.3390/molecules22030386

**Published:** 2017-03-01

**Authors:** Yang Yang, Tian Gao, Mengjun Xu, Jie Dong, Hanxiao Li, Pengfei Wang, Gezi Li, Tiancai Guo, Guozhang Kang, Yonghua Wang

**Affiliations:** 1 The Collaborative Innovation Center of Henan Food Crops, Henan Agricultural University, Zhengzhou 450002, China; yangyang91@henau.edu.cn (Y.Y.); gaotian1211@henau.edu.cn (T.G.); wangpf@henau.edu.cn (P.W.); 2 The National Key Laboratory of Wheat and Maize Crop Science, Henan Agricultural University, Zhengzhou 450002, China; mengjunxu920304@henau.edu.cn (M.X.); dongjie@henau.edu.cn (J.D.); gtcwheat@henau.edu.cn (T.G.); 3 The National Engineering Research Centre for Wheat, Henan Agricultural University, Zhengzhou 450002, China; xxhong@henau.edu.cn (H.L.); ligezi@henau.edu.cn (G.L.)

**Keywords:** AGPase, plastidial small subunit, starch, *Triticum aestivum* L.

## Abstract

ADP-glucose pyrophosphorylase (AGPase), the key enzyme in starch synthesis, consists of two small subunits and two large subunits with cytosolic and plastidial isoforms. In our previous study, a cDNA sequence encoding the plastidial small subunit (TaAGPS1b) of AGPase in grains of bread wheat (*Triticum aestivum* L.) was isolated and the protein subunit encoded by this gene was characterized as a truncated transit peptide (about 50% shorter than those of other plant AGPS1bs). In the present study, TaAGPS1b was fused with green fluorescent protein (GFP) in rice protoplast cells, and confocal fluorescence microscopy observations revealed that like other AGPS1b containing the normal transit peptide, TaAGPS1b-GFP was localized in chloroplasts. TaAGPS1b was further overexpressed in a Chinese bread wheat cultivar, and the transgenic wheat lines exhibited a significant increase in endosperm AGPase activities, starch contents, and grain weights. These suggested that TaAGPS1b subunit was targeted into plastids by its truncated transit peptide and it could play an important role in starch synthesis in bread wheat grains.

## 1. Introduction

Starch is one of the primary plant carbohydrate reserves in higher plants and it is composed of two different types of glucose (Glc) polymers: amylose (a linear polymer composed of α-1,4-glucosidic link chains) and amylopectin (a highly branched glucan with α-1,6 glucosidic bonds that connect linear chains) [1]. Adenosine diphosphate glucose pyrophosphorylase (AGPase, EC 2.7.7.27) converts glucose-1-phosphate (G-1-P) and ATP to adenosine diphosphate glucose (ADP-Glc). Granule-bound starch synthase (GBSS, EC 2.4.1.242) is mainly involved in amylose production, whereas soluble starch synthase (SS, EC 2.4.1.21), starch branching enzyme (BE, EC 2.4.1.18), and starch debranching enzyme (DBE) (isoamylase, ISA, EC 3.2.1.68; pullulanase, PUL, EC 3.2.1.41) work together, with distinct roles to catalyze the amylopectin synthesis [2,3]. Disproportionating enzyme (DPE, EC 2.4.1.25) and phosphorylase (PHO, EC 2.4.1.1) have been thought to function in the initiation step of starch biosynthesis, although their precise mechanisms of action remain obscure [2].

AGPase is thought to be the key enzyme of starch biosynthesis, because ADP-Glc catalyzed by this enzyme is the precursor for the synthesis of both amylose and amylopectin [1]. In transgenic *Arabidopsis thaliana* plants expressing a bacterial β-glucuronidase (*GUS*) gene under the control of the AGPS gene promoter, the expression patterns of *GUS* gene followed closely the AGPS protein location and starch production in plant tissues/cells, indicating the essential function of AGPase subunits in starch synthesis in higher plants [4]. AGPase is a homotetramer (α_4_) in bacteria, whereas it is a heterotetramer in higher plants, in which each molecule carries two smaller subunits (AGPS or SSU = α_2_) and two slightly larger subunits (AGPL or LSU = β_2_). Thus, AGPase in higher plants is an α_2_β_2_ heterotetramer [5]. AGPSs form a homotetrameric enzyme exhibiting normal catalytic properties, AGPLs provide the enzymatic regulatory properties, which increase the allosteric response of AGPSs to 3-phosphoglyceric acid (3-PGA) and inorganic phosphate (Pi), and these two subunit types are essential for normal AGPase activity [6]. In higher plants, starch synthesis takes place inside plastids (the leaf chloroplasts in photosynthetic organs and the grain amyloplasts in non-photosynthetic starch-storing organs) [7]. In graminaceous species, AGPases have cytosolic and plastidial isoforms and these two types function independently to produce starch. Thus, there are four types of AGPase subunits in plant cells: cytosolic AGPS, cytosolic AGPL, plastidial AGPS, and plastidial AGPL. In plant leaf tissue, AGPase generally occurs only in plastids. In the grain endosperm of most cereal crops, however, the predominant form of AGPase occurs in the cytosol and only a minor form resides in the amyloplasts [8]. For instance, cytosolic AGPase activity represents approximately 85% and 95% of the total AGPase activity in developing endosperms of barley and maize, respectively [9,10]. These account for the importance of cytosolic AGPase for starch synthesis in these species. These have also been demonstrated by low-starch mutants of barley and maize which lack the cytosolic form of AGPase [11].

Together with maize and rice, bread wheat is one of three most important crops globally, and it provides approximately 20% of the total food calories and proteins for the human diet [12]. Unlike in barley and maize, however, the proportion of plastidial AGPase activity is higher in wheat endosperm, and approximately 30% of endosperm AGPase activity in wheat [13] is plastidial compared with about 15% in barley [14] and 5% in maize [9]. These suggest that, besides cytosolic AGPase, plastidial AGPase also plays important role in endosperm starch synthesis in bread wheat. In rice, the model species of monocotyledonous plants, the AGPase gene family consists of two *AGPS* genes, *OsAGPS1* and *2*, and four *AGPL* genes, *OsAGPL1-4*. *OsAGPS2* generates two transcripts, *OsAGPS2a* and *OsAGPS2b*. OsAGPS2b and OsAGPL2 subunits are localized to the cytosol, whereas the other subunits present in the plastids. In barley and bread wheat, two genes encoding the AGPSs (*AGPS1* and *AGPS2*) of AGPase have been identified. *AGPS2* gives rise to a single transcript encoding a plastidial AGPS. Similar with *OsAGPS2* gene, *AGPS1* in these two species gives rises to two transcripts by use of the alternative first exons. One of the alternative first exons, exon1b (*AGPS1b*), encodes a transit peptide for targeting its precursor protein to plastids. The other first exon, exon 1a, is shorter than exon 1b and does not contain a predicted transit peptide. This suggests that one of the proteins encoded by *AGPS1* is cytosolic (*AGPS1a*) and the other is plastidial (*AGPS1b*). The amino acid sequences of *AGPS1a* and *b* subunits in these two species are identical over 90% of their length, and differ only at their N termini. The predicted N-terminal domain unique to the plastidial AGPS1 in bread wheat and barley contains a transit peptide, whereas the predicted N-terminal domain of the cytosolic protein is shorter than a typical transit peptide and lacks a consensus transit peptide cleavage site [7,10].

In our previous study, a cDNA sequence (GenBank accession No. EU586278) encoding one transcript of *TaAGPS1b* was isolated from grains of some Chinese bread wheat cultivars. Compared with another *TaAGPS1b* transcript in bread wheat, this isolated transcript lacked a long fragment (117 bp) at its 5′ terminal, resulting in a truncated transit peptide [15]. The predicated transit peptide of this AGPase subunit merely contained 25 amino acids, considerably shorter than those of other plant *AGPS1bs* (54–70 amino acids). Although it lacked a long fragment at its 5′ terminal, the predicated transit cleavage site (R|A) of this TaAGPS1b subunit was the same as those of other cereal *AGPS1bs*. Their amino acid residues behind the transit peptides also shared high similarities. Thus, we speculated that the capacity of the transit peptide for our isolated TaAGPS1b subunit, and the catalytic function of the AGPase enzyme, composed by this plastidial small subunit and the related AGPase plastidial large subunit, could not be changed. To test this speculation, in the present study, TaAGPS1b (EU586278) was fused with the green fluorescent protein (GFP) and then transformed into rice protoplasts for subcellular localization analysis. Its overexpression vector was constructed and transformed into a bread wheat cultivar to measure starch contents for its functional analysis.

## 2. Results and Discussion

### 2.1. TaAGPS1b-EU586278 Subunit with the Truncated Transit Peptide Could Be Located in Plastid

Transit peptide, as a kind of signal peptide, carries the information required for targeting to the correct organelle [16]. Most plastidial proteins are encoded by the nuclear genome, synthesized in the nucleocytoplasm as preproteins with N-terminal extensions (transit peptides, targeting peptides or presequences) that are required for protein import into the organelles, and in most cases, the targeting peptides are removed by intraorganellar proteases during or shortly after import [17]. In the photosynthetic tissues of the majority of higher plant species, the synthesis of ADP-Glc, which is catalyzed by plastidial AGPase enzyme, occurs entirely within the chloroplasts, and preproteins of AGPS (SSU) and AGPL (LSU) are synthesized in nuclear genome and directly imported into the chloroplasts by their transit peptides [7].

**Figure 1:**
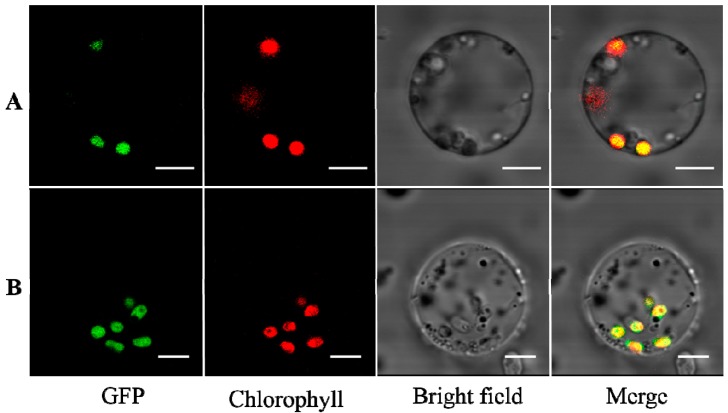
Subcellular localization of FJ643609-GFP (**A**) and EU586278-GFP (**B**) fusion proteins into rice protoplasts. Notes: (1) Full-length *TaAGPS1b-FJ643609* and *-EU586278* cDNA sequences were fused in-frame to GFP, respectively, and expressed under the control of the CaMV35S promoter. (2) All images were recorded at the same sensitivity and scale. (3) Each set of four images shows the same cell. Bars = 5 μm.

*TaAGPS1b-EU586278*, a transcript encoding plastidial AGPS characterized with a truncated transit peptide, was isolated in our previous study [15]. In this study, the subcellular localization of TaAGPS1b-EU586278 subunit was examined in rice protoplasts using FJ643609-GFP as positive control. Our data indicated that, like the TaAGPS1b-FJ643609 transcript, TaAGPS1b-EU586278 was also located in chloroplasts (Figure 1). This suggested that the targeting function of this truncated transit peptide in TaAGPS1b-EU586278 subunit could not be changed and this truncated transit peptide could target TaAGPS1b-EU586278 preproteins into plastids (chloroplasts in leaf cells or amyloplasts in grain cells). Many transit peptides contain three distinct regions: an uncharged N-terminal domain of ~10 residues beginning with methionine (Met) and alanine (Ala) and terminating with a glycine (Gly) or proline (Pro), a central domain lacking acidic residues but enriched in serine (Ser) or threonine (Thr) and, finally, a C-terminal domain enriched in arginine (Arg) [18]. The N- and the C-terminal domains can contain α-helical structure underlying the structural preferences of a given peptide sequence, however, the central domain often forms a random coil, which can act as a recognition motif for preprotein import [18]. Compared to other AGPS1b subunits, the lacked fragment of TaAGPS1b-EU586278 was located at the central domain [15]. Our results suggested that the lacked fragment of the TaAGPS1b-EU586278 transit peptide could not be recognition motif and its lack could not change the targeting function of the transit peptide.

The cleavage site (R|A) of TaAGPS1b-EU586278 transit peptide, which was predicted with TargetP software in our previous study [19], was the same as other AGPS1bs, and their amino acid sequences behind the cleavage sites were highly identical, allowing us to speculate that after TaAGPS1b-EU586278 preprotein was directed into plastids and its transit peptide was cleaved off, the functional amino acid sequence of its matured TaAGPS1b subunit was not changed [15]. However, the reliable predication [reliability coefficient (RC), RC = 3] on the transit peptide of TaAGPS1b-EU586278 was lower than those (RC = 1−2) of other AGPS1bs, suggesting that this prediction needed to be further confirmed.

### 2.2. Function of TaAGPS1b-EU586278 with the Truncated Transit Peptide Could Not Be Changed

Measuring the activities of the enzymes encoded by the target genes for their functional studies by in vitro or vivo protocols should be quick and effective. Because both two homo-AGPLs and two homo-AGPSs in higher plants compose a heterotetramer AGPase and only AGPS has no enzymatic activity without regulatory properties from AGPL [5], it is difficult to study enzymatic function of alone AGPS protein encoded by *TaAGPS1b-EU586278* transcript although it could be easily extracted in vitro (e.g., in *E. coli*). More, it could be difficult to solely isolate the especial AGPase enzyme composed of AGPL and TaAGPS1b-EU586278 subunit in vivo and measure its activity because the matured TaAGPS1b-EU586278 subunit containing the truncated transit peptide could be the same as other matured TaAGPS1s containing the normal transit peptides. Moreover, it is also difficult to separately quantify the enzymatic activity or the quantity of every isoform in plant cells because most of starch synthesis enzymes have multiple isoforms or subunits and they are usually labile [20]. In our opinion, it could be feasible to evaluate the function of *TaAGPS1b-EU586278* using transgenic experiments.

**Figure 2:**
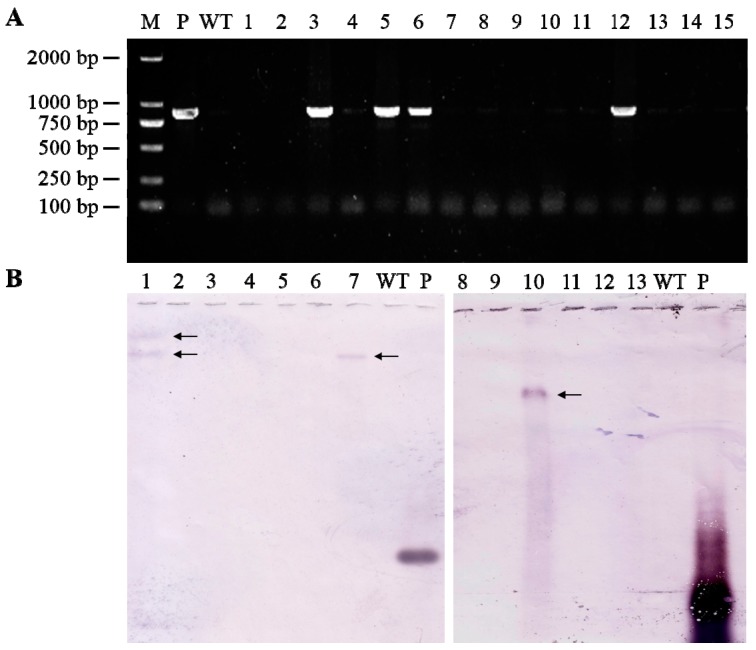
Analyses of wheat lines transformed with pWM101-EU586278 using PCR and southern blot methods. (**A**) PCR analysis of genomic DNA with *Hyg* gene. M, DL2000 marker; P, plasmid pWM101-EU586278 (positive control); WT, the untransformed wild plant (negative control); lines 1–15, wheat plants transformed with pWM101-EU586278; (**B**) Southern blot. Lines 1–13, thirteen randomly selected PCR positive lines; WT, the untransformed wild plant (negative control); P, the purified probe product (positive control).

**Figure 3:**
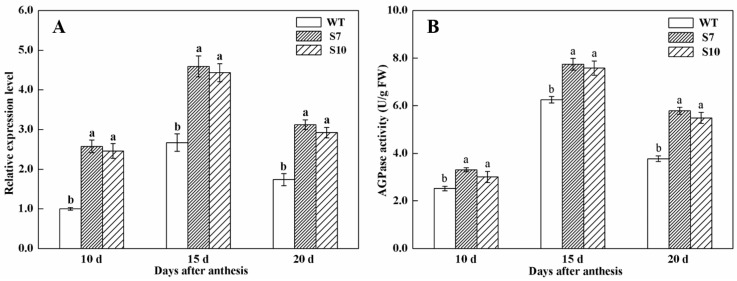
Transcript levels of *TaAGPS1b-EU586278* (**A**) and AGPase activities (**B**) in endosperm of the developing grains of WT and transgenic wheat lines. Notes: (1) Transcript levels at 10, 15 and 20 days after anthesis were measured by qPCR using β-actin gene as internal control. (2) WT, the untransformed wild plant; S7 and S10, the two independent TaAGPS1b-EU586278 T_3_ transgenic wheat lines. (3) Each value is the mean ± SD of three independent biological replicates. Different letters represented statistical significance at *p* < 0.05.

**Figure 4:**
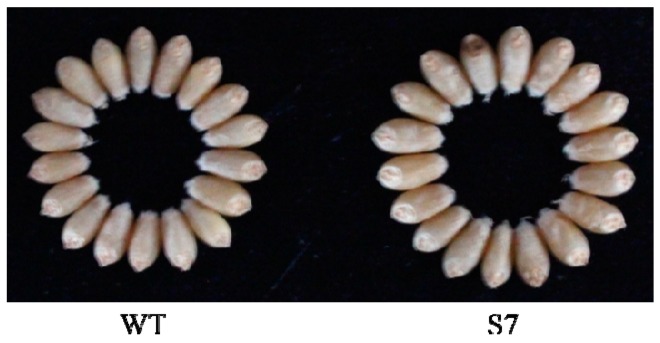
Comparison of phenotype of mature grains between WT and the representative transgenic wheat lines. Eighteen grains were randomly selected in WT and S7 line, respectively.

In this study, the *TaAGPS1b-EU586278* transcript was overexpressed in wheat plants, and transgenic wheat plants were identified using PCR and southern blot analysis (Figure 2). These data indicated that these three transgenic lines overexpressing *TaAGPS1b-EU586278* gene were identified, and of these, two (S7 and S10) were inherited by the progeny in a single copy and were used for the following experiments. Compared with WT, the transcript levels of *TaAGPS1b-EU586278* in these two transgenic wheat lines significantly increased at 10, 15 and 20 days after anthesis (Figure 3A, Figure S2). AGPase activities also showed similar changes in these transgenic wheat lines (Figure 3B). At mature stage, starch contents in the harvested grains of S7 and S10 transgenic wheat lines were significantly increased by 14.6% and 11.5% higher than those in WT, respectively (Table 1). The increased grain starch contents in transgenic wheat lines could result in the remarkably improved grain sizes, kernel weights per grain and individual spike weights (Table 1, Figure 4). These suggested that TaAGPS1b-EU586278 preprotein could form normal mature AGPS1 subunits and combine with TaAGPL to compose the heterotetramer AGPase enzyme for starch synthesis after it was directed into grain amyloplasts and its truncated transit peptide was cleaved off.

In most bread wheat planting regions of China, genetic improvement in grain yield in recent twenty years has mainly been focused on achieving increased kernel weights [21]. However, wheat grain filling is seriously inhibited by frequent abiotic and biotic stresses, and kernel weights of most wheat cultivars in China rarely achieve their own maximal potential. In the present study, transgenic wheat lines overexpressing *TaAGPS1b-EU586278* showed increased kernel weights without remarkable changes in phenotype (plant height, leaf number, spike shape, etc), growth and development stages (seedling, overwintering, re-greening, jointing, anthesis, filling, maturity, etc) (data no shown), and other two yield components (spike number per plant and grain number per spike) (Table 1). These suggested that the transgenic wheat lines overexpressing *TaAGPS1b-EU586278* transcript could have potential applications to develop a high-yield wheat cultivar with high starch and kernel weight.

Modifying the allosteric properties (the inhibited insensitivity to Pi) of AGPase heterotetramers could increase grain weight by increasing grain number per plant without changing starch content [22,23,24]. For example, Giroux and his colleagues exploited site-specific mutagenesis system to create short insertion mutations (3 or 6 bp) of maize *AGPL1* gene (*Sh2*) named *Rev6* and these lines had 11%–18% greater individual grain weight by increasing grain number per spike, but without changing starch content or single grain weight [22]. They proposed that the decreased sensitivity of AGPase to allosteric inhibition (Pi) might effectively mobilize resources to adjacent later-setting grains, enhancing their ability to avoid abortion [23]. According to data shown in this study (Table 1), however, overexpression of one or more AGPase subunits could improve grain weight by increasing starch content and per seed weight, but without changing the grain number per spike [25,26,27,28]. For instance, Li and his colleagues reported that overexpression of either the *Sh2* gene, or *Bt2* gene coding for maize AGPS1 and AGPL1 subunits, did not affect the number of grain rows or number of grains per ear, whereas resulted in enhanced seed weight and starch content, and they proposed that this might be related to the enhanced photosynthetic capability and carbon metabolite levels, or the formed more tetramers of AGPase [26]. These results implied that the increased grain yield by regulating AGPase subunit expression could be complex in cereals. To deeply explore the mechanism of the TaAGPS1b-EU586278 subunit in regulating starch synthesis, our following experiments would be performed to determine the number of AGPase tetramers, the rates of photosynthesis, and the contents of carbohydrates in the transgenic wheat plants.

## 3. Materials and Methods

### 3.1. Subcellular Localization of TaAGPS1b Subunit with the Truncated Transit Peptide

Two bread wheat transcripts encoding two AGPS1b subunits with the truncated transit peptide (GenBank accession No. EU586278) and the untruncated transit peptide (GenBank accession No. FJ643609) were isolated in our previous study [15]. In this study, their coding sequences (CDSs) without stop codon were separately amplified with the primers (Table S1). The amplified fragments were digested with the *Kpn* I and *Bam*H I endonucleases and inserted into the HBT-sGFP vector [29] containing the green fluorescent protein (GFP) gene to construct two HBT-*TaAGPS1b*-GFP vectors (EU586278-GFP and FJ643609-GFP) (Figure S1). Next, these two GFP fusion vectors were separately transformed into rice protoplasts as described by Zhang and his colleagues [30]. The rice protoplasts were incubated at room temperature for 16 h, and fluorescence signals of EU586278-GFP and FJ643609-GFP were measured with a laser-scanning confocal microscopy (FV10-ASW, Olympus, Tokyo, Japan). GFP fluorescence was imaged at an excitation wavelength of 488 nm, and chlorophyll autofluorescence was imaged at an excitation wavelength of 560 nm.

### 3.2. Identification of Transgenic Wheat Plants Expressing TaAGPS1b-EU586278 Transcript

CDS sequence of EU586278 transcript containing stop codon was amplified using the primers 5’-ATGGCGATGGCCGCGGCC-3’ (sense) and 5’-TCATATGACTGTTCCACTAGGGAGT-3’ (antisense). The plant expression vector pWM101, which contains the selective marker gene of *hygromycin* (*Hyg*) controlled by the 35 S promoter and 35 S polyA terminator, was used as the backbone for construction [31]. Then, this CDS was inserted into pWM101 with *Bam*H I and *Xba* I endonucleases to construct plasmid pWM101-*TaAGPS1b* overexpression vector (pWM101-EU586278) (Figure S2). Transgenic wheat progenies were generated by *Agrobacterium* inoculum to the basal portions of wheat seedlings as described in previous studies [28,32]. Coleoptiles of seedlings with 2–4 cm height from wheat cultivar Yumai 34 were used to transform pWM101-EU586278 vector. In this cultivar, transcript of *TaAGPS1b* gene was also amplified and sequenced, and it was found that its transcript is identical to that of EU586278, suggesting that the transit peptide of TaAGPS1b subunit encoding this transcript is truncated in this wheat cultivar [15]. After the transformed wheat seedlings had three to four leaves, resistance selection was performed by insufflation of the seedlings with solution containing 180 mg·L^−1^ Hyg. The resistant seedlings (T_0_) were planted in the Experimental Farm of Agricultural Faculty, Henan Agricultural University (34°N, 113°E, and 52 m elevation). Seedlings of T_1_ and T_2_ parents were further selected using both herbicide selection and PCR analysis, the Hyg-resistant and PCR-positive seedlings were self-pollinated and their seedlings were harvested according to our previous methods [28]. One of Hyg-resistant, PCR analysis-positive and homozygous T_2_ plants was randomly sampled for southern blot analysis and southern blot-positive wheat plants were allowed to produce T_3_ seeds. These T_3_ seeds were randomly sowed, grown, and identified for phenotypic analysis. Homozygous and PCR-positive T_3_ progenies were selected for physiological and biochemical analysis.

At every transgenic wheat parent, genomic DNA from every Hyg-resistant parents was extracted from young leaves using the CTAB method for PCR analysis [33], primers 5’-ATCGTTATGTTTATCGGCACT-3’ (sense) and 5’-CGGTCGGCATCTACTCTATT-3’ (antisense) from the Hyg resistance gene were used and a fragment (863 bp) was amplified. The PCR amplification programs were as follows: 95 °C for 5 min, then 32 cycles of 30 s at 95 °C, 40 s at 53.5 °C, and 60 s at 72 °C.

At T_2_ parents, genomic DNA samples (30 μg) from Hyg-resistant and PCR analysis-positive wheat plants were digested for 2 h at 37 °C with *Eco*R I endonuclease, which has no cut site in T-DNA region. Digested wheat genomic DNA was separated on to 1% (*w*/*v*) agarose gels and transferred to Hybond N^+^ nylon membranes. The above PCR amplified fragments were labeled with digoxigenin (DIG)-dUTP as the probes. Southern blot was conducted at 68 °C for overnight and hybridization and detection were performed according to our previous study [28].

### 3.3. Transcript Levels of TaAGPS1b-EU586278 Gene in Transgenic Wheat Lines

At T_3_ parents, total RNA from the endosperm of WT and transgenic wheat lines at 10, 15, and 20 days after anthesis was isolated with the extraction buffer (50 mM Tris pH 9.0, 200 mM NaCl, 1% (*w*/*v*) sarcosyl, 20mM EDTA, and 5 mM DTT) to remove starch, and further treated with RNase-free DNase I (Takara Biotechnology Co., Ltd., Dalian, China) to remove contaminating genomic DNA. 2 μg isolated RNA was used for cDNA synthesis using the PrimeScript™ RT Reagent Kit (Perfect Real Time) (Takara Biotechnology Co., Ltd.) and real-time quantitative RT-PCR (qPCR) was performed for determination of *TaAGPS1-EU586278* transcripts using the SYBR Premix Ex Taq Kit (Takara Biotechnology Co., Ltd.) according to the manufacturer’s instructions. To minimize sample variation, mRNA expression of the target gene was normalized relative to wheat *glyceraldehydes 3-phosphate dehydrogenase* (*GAPDH*) (GenBank accession No. EF592180) and β-actin (GenBank accession No. AB181991) genes expression levels. Primers used for qPCR are listed in Table S1.

### 3.4. Assays of AGPase Activity in Grains of Transgenic Wheat Lines

Activities of AGPase enzyme were assayed for the same transgenic wheat lines for analysis of the above transcript levels of *TaAGPS1b-EU586278* at the same sampling stages using the method described previously [26]. Embryo and aleurone layer were manually removed from harvested grains and the remained endosperm (about 1 g) was ground on ice in 5 mL of extraction buffer (50 mM HEPES, pH 7.5; 50 mM MgCl_2_; 10 mM EDTA; 12.5% (*v*/*v*) glycerol; and freshly added 5% (*m*/*v*) PVP, and 50 mM β-mercaptoethanol). After 15 min centrifugation (10, 000 *g*, 4°C), 50 μL of enzyme supernatant was mixed with 250 μL of enzyme reaction mixture (100 μL of 5 mM ADPG, 50 μL of 50 mM MgCl_2_, and 100 μL of 50 mM HEPES-NaOH) to the final concentrations of 1.7 mM ADPG, 8.3 mM MgCl_2_, and 16.7 mM HEPES-NaOH and incubated for 10 min at 30 °C. Then, 100 μL of 20 mM Pi was added to initiate the reaction, and the mixture was incubated at 30°C for 15 min. The reaction was stopped by boiling for 1 min, and cooled to room temperature. Total 500 μL of reaction solution (100 μL of 6 mM NADP^+^, 50 μL of 1.5 U/mL P-glucomutase, 50 μL of 5 U/mL glucose-6-phosphate dehydrogenase, and 300 μL of 50 mM HEPES-NaOH (pH 7.5)) was added. After incubation, the absorbance of the solution was measured at 340 nm with a UV-2600 spectrophotometer (UNICO (Shanghai) Instruments Co., Ltd., Shanghai, China]. One unit was defined as amounts of AGPase that catalyzed production of 1 nmol NADPH per min.

### 3.5. Analysis of Starch Content and Yield Characteristics between WT and Transgenic Wheat Lines

Mature grain harvested from wild and T_3_ transgenic wheat lines were used for analysis on starch content and yield characteristics. Harvested grains were weighted after drying to equivalent moisture content. The starch contents were measured and calculated according to methods of Zhao and his colleagues [34]. The yield characteristics of spike number, grain number per spike, kernel weight per grain and grain weights of individual spike were evaluated according to our previous study [28].

### 3.6. Statistical Analysis

Three biological replications were performed, and all data are the mean ± standard deviation (SD) of three independent experimental replicates. Data were statistically analyzed using one-way analysis of variance (ANOVA), and means were compared by using the Duncan's multiple range test at *p* < 0.05 level by SPSS (version 20.0) statistical software. 

## 4. Conclusions

TaAGPS1b (EU586278), a wheat AGPase plastidial small subunit with a truncated transit peptide, was proved to be localized in chloroplasts and its overexpression transgenic wheat plants exhibited the improved AGPase activities, starch contents, and grain weights, indicating its important role in starch synthesis. 

## Figures and Tables

**Table 1:** Comparisons of grain characterizations between WT and transgenic wheat lines in the field.

Lines	Spike Number (per Plant)	Grain Number (per Spike)	Kernel Weight per Grain (mg)	Weight of Individual Spike (g)	Starch Content (mg/Grain)
WT	6.3 ± 0.3a	23.0 ± 0.6a	53.28 ± 0.33b	1.23 ± 0.03b	34.77 ± 0.64b
S7	7.0 ± 0.6a	23.3 ± 0.3a	59.02 ± 0.61a	1.38 ± 0.01a	39.86 ± 0.52a
S10	6.7 ± 0.3a	23.7 ± 0.3a	57.12 ± 1.47a	1.35 ± 0.16a	38.77 ± 1.09a

Data were mean ± SD (*n* = 3). Different letters represented statistical significance at *p* < 0.05.

## Supplementary Materials

Supplementary materials are available online.
