# Supplementary material for: Functional Analysis of a Wheat AGPase Plastidial Small Subunit with a Truncated Transit Peptide
Source: Molecules. 2017 Mar 1;22(3):386. doi: 10.3390/molecules22030386 (PMC6155376; doi:10.3390/molecules22030386)
Supplement: Supplementary file 1 [file molecules-22-00386-s001.pdf]

Table S1. Primers used in this study.

Types	Primer names	Primer sequences (5' to 3')
FJ643609-GFP	GFP-F	CGGGGTACCATGGCGATGGCCG
vector	GFP-R	CGCGGATCCTATGACTGTTCCAC
EU586278-GFP	GFP-F	CGGGGTACCATGGCGATGGCCG
vector	GFP-R	CGCGGATCCTATGACTGTTCCAC
qPCR	qPCR-F	AAGATCCTGATCCCTCCG
	qPCR-R	CTAGTCCCTGCACCACCT
	β-actin-F	AGCGGTCGAACAACTGGTA
	β-actin-R	AAACGAAGGATAGCATGAGGAAGC
	GAPDH-F	TTTTCACCGACAAGGACA
	GAPDH-R	AAGAGGAGCAAGGCAGTT

Notes: F, forward primer, R, reverse primer.

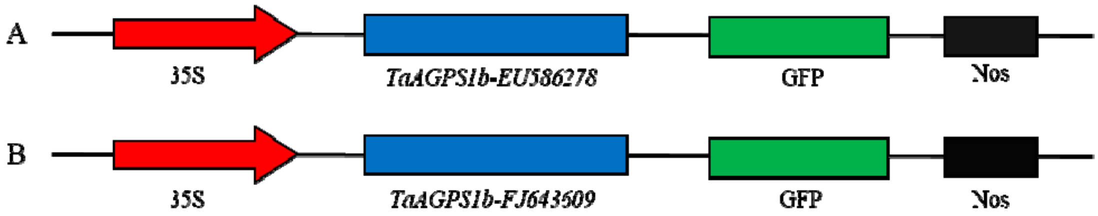

Figure S1. The plasmid constructs of TaAGPS1b-EU586278 (A) and TaAGPS1b-FJ643609 (B) for subcellular localization. 35S, CaMV 35S promoter; GFP, the green fluorescent protein gene; Nos, Nos terminator.

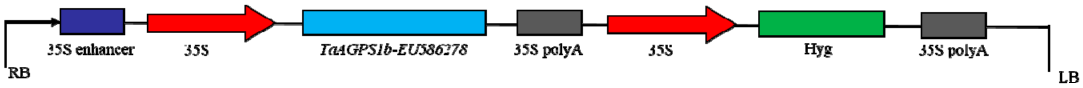

Figure S2. Construction of an expression cassette pWM101-EU586278 in a binary vector was performed as described in the Materials and Methods. 35S, CaMV 35S promoter; *TaAGPS1b-EU586278*, CDS sequence of *EU586278* transcript; Hyg, hygromycin resistance gene; RB, right T-DNA border; LB, left T-DNA border.

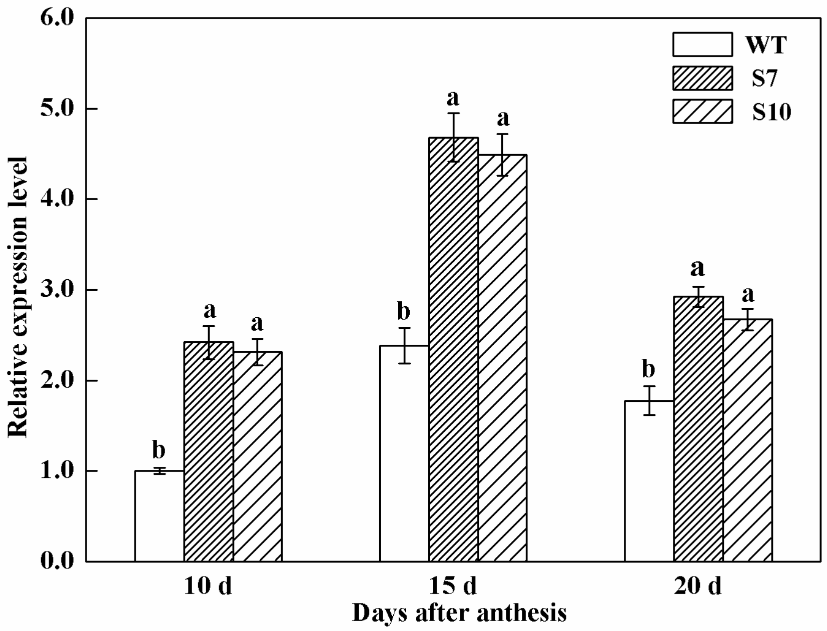

Figure S3. Transcript levels of *TaAGPS1b-EU586278* in endosperm of the developing grains of WT and transgenic wheat lines. Notes: (1) Transcript levels at 10, 15 and 20 days after anthesis were measured by qPCR using *GAPDH* gene as internal control. (2) WT, the untransformed wild plant; S7 and S10, two independent TaAGPS1b-EU586278 T_3 transgenic wheat lines. (3) Each value is the mean ± SD of three independent biological replicates. Different letters represented statistical significance at $P < 0.05$.
